# Cognitive frailty is a robust predictor of falls, injuries, and disability among community-dwelling older adults

**DOI:** 10.1186/s12877-021-02525-y

**Published:** 2021-10-25

**Authors:** Nurul Fatin Malek Rivan, Devinder Kaur Ajit Singh, Suzana Shahar, Goh Jing Wen, Nor Fadilah Rajab, Normah Che Din, Hazlina Mahadzir, Mohd Zul Amin Kamaruddin

**Affiliations:** 1 Nutritional Sciences Programme and Centre for Healthy Ageing and Wellness (H-CARE), Faculty of Health Sciences, Universiti Kebangsaan Malaysia, Jalan Raja Muda Abdul Aziz, 50300 Kuala Lumpur, Malaysia; 2 Physiotherapy Programme & Centre for Healthy Ageing and Wellness (H-CARE), Universiti Kebangsaan Malaysia, Jalan Raja Muda Abdul Aziz, 50300 Kuala Lumpur, Malaysia; 3 Dietetics Programme and Centre for Healthy Ageing and Wellness (H-CARE), Faculty of Health Sciences, Universiti Kebangsaan Malaysia, Jalan Raja Muda Abdul Aziz, 50300 Kuala Lumpur, Malaysia; 4 Biomedical Science Programme, Centre for Healthy Ageing and Wellness (H-CARE), Faculty of Health Sciences, Universiti Kebangsaan Malaysia, Jalan Raja Muda Abdul Aziz, 50300 Kuala Lumpur, Malaysia; 5 Health Psychology Programme and Centre of Rehabilitation Science, Faculty of Health Sciences, Universiti Kebangsaan Malaysia, Jalan Raja Muda Abdul Aziz, 50300 Kuala Lumpur, Malaysia; 6 Internal Medicine & Geriatric Department, Pusat Perubatan Universiti Kebangsaan Malaysia, Jalan Yaacob Latif, Bandar Tun Razak, Batu 9 Cheras, 56000 Kuala Lumpur, Malaysia; 7 Centre for Healthy Ageing and Wellness (H-CARE), Faculty of Health Sciences, Universiti Kebangsaan Malaysia, Jalan Raja Muda Abdul Aziz, 50300 Kuala Lumpur, Malaysia

**Keywords:** Cognitive frailty, Falls, Injuries, Disability, Incidence, Predictors, Older adults

## Abstract

**Background:**

Cognitive frailty, a combination of physical frailty and cognitive impairment, is associated with functional decline in older adults. However, there is limited information if cognitive frailty predicts the incidence of falls, injuries, and disability. In this study, we aimed to determine the ability of cognitive frailty in predicting the incidence of falls, injuries and disability among multi-ethnic older adults in Malaysia at 5 years follow-up.

**Methods:**

In this prospective cohort study, a total of 400 participants aged 60 years and above were successfully followed up at 5 years. Participants’ socio-demographic, medical history, psycho-social, physical, cognitive and dietary intake information was obtained. Cognitive frailty was defined as comorbid physical frailty (> 1 Fried criteria) and mild cognitive impairment (Petersen criteria). Univariate analysis was performed for all variables, followed by hierarchical binary logistic regression (BLR) analysis to identify the ability of CF in predicting the incidence of falls, injuries, and disability. The significant value was set at *p* < 0.05.

**Results:**

Cognitive frailty was found to be associated with greater risk of adverse consequences after adjusting for covariates. Both cognitive frailty (Adjusted Odd ratio (Adj OR) = 2.98, 95% confidence interval (CI): 1.78–4.99, *p* < 0.05) and physical frailty (Adj OR = 2.88, 95% CI: 1.19–6.99, p < 0.05) were significant predictors of incidence of falls. Risk of injuries was also significantly increased with the presence of cognitive frailty (Adj OR = 3.06, 95% CI: 1.23–7.60, p < 0.05) and physical frailty (Adj OR = 3.04, 95% CI: 1.75–5.28, p < 0.05). In addition, cognitive frailty (Adj OR = 5.17, 95% CI: 1.11–24.21, p < 0.05) and physical frailty (Adj OR = 4.99, 95% CI: 1.11–22.57, p < 0.05) were shown to significantly predict the incidence of disability among older adults.

**Conclusion:**

Cognitive frailty is a robust predictor of falls, injuries, and disability in older adults. Possible early multi-domain preventive and management strategies of cognitive frailty that contribute to adverse consequences are required to decrease further functional decline and promote independence in older adults.

## Introduction

Frailty, loss of functional reserve and resistance to internal and external stressors can lead to adverse health-related consequences, particularly falls, related injuries, disability, hospitalization, institutionalization and increased mortality [1–3]. Cognitive impairment has also been shown to have an association with frailty [4], falls and disability incidence [5]. Impairments in physical and cognitive function increase the risk of related adverse consequences in older adults [6–8]. Based on this, the cognitive frailty (CF) concept was introduced and is characterized by the simultaneous presence of both physical frailty (PF) and mild cognitive impairment (MCI) [9].

Global prevalence rate of CF is between 1.0 to 4.4% among older adults [7, 10, 11]. In community-dwelling Malaysian older adults, the CF incidence rate of 7.1 per 100 person-years has been reported [12]. We speculate a higher incidence rate of CF in older adults residing at institutions or nursing homes. In addition, a higher risk of falls, related injuries and disability incidence is deduced with CF due to its combined risk of both PF and MCI. We have also previously reported the combined prevalence of physical frailty and mild cognitive impairment, where 39.6% of our older population had CF [13].

Falls, related injuries and disability as adverse consequences of the many health-related issues in older adults is a public health concern globally. The number of falls is projected to rise, parallel with the increase in the number of older adults over the age of 80 years [14]. Falls prevalence among Malaysian community-dwelling older adults is estimated to be approximately 15 to 27% [15, 16]. The identified falls risk factors included declined muscle strength, poor self-rated health, urinary incontinence and chronic diseases (diabetes, arthritis) [17]. Falls in older adults may cause serious injuries such as fractures, joint dislocations, and head injuries leading to dependency, poor quality of life and disability [18, 19].

In Malaysia, the prevalence of falls and falls-related injuries were 4.07 and 5.8% among community-dwelling older adults [6, 20]. Furthermore, the disability-adjusted life years (DALYs) rate reported in the Global Burden Diseases (GBD) in 22 European countries showed a slight decline over time from 2245 DALYs per 100,000 in 1990 to 2227 DALYs per 100,000 in 2017 [21]. As the DALYs rate decreases, there is a shifting trend towards years lived with disability (YLD), where it becomes the primary driver of fall-related injury DALYs in older adults instead of years of life lost (YLL) rates [21]. However, these incidence data on fall-related injury were based on the Western countries, which may not represent the Asian older population.

In accordance with the evidence, disability may both be an adverse consequence or cause of falls [18]. Some of the indicators of disability progression, such as mobility and activities of daily living (ADL) was significant markers of frailty [1, 8]. Previous studies have also reported that cognitive impairment is associated with reduced ADL and should be a prognostic marker in predicting the progression of disability [5]. Moreover, one of the risk factors of CF was physical disability, indicated by lower performance in ADL [13].

Although PF and MCI are well-known risk factors for geriatric syndromes, the concomitant effects of CF in predicting the incidence of falls, injuries and disability are presently unclear. The associations between CF, fall and fall-related fractures have been reported in a cross-sectional study [22]. However, these studies design limits its interpretation in the causal relationship [18, 23]. Besides, evidence shows that older adults with CF had a greater risk of hospitalization, dementia, falls and disability in previous longitudinal studies [7, 10, 24, 25]. However, the CF definition proposed in these studies had a low prevalence of CF in community-based settings (1.0–12.0%), and those with pre-frailty were excluded [7, 10, 24, 25]. To address this gap, we included older adults with pre-frailty and frailty with MCI to detect a sufficient number of at risk older adults and improve its predictive validity for early prevention of adverse health outcomes. The aim of this study was to determine the ability of CF in predicting the incidence of falls, injuries and disability among multi-ethnic older adults in Malaysia at 5 years follow-up.

## Methodology

### Study design and participants

The participants and study design of this study have been reported previously [13] and is a five years follow up of the Longitudinal Study on Neuroprotective Model for Healthy Longevity (LRGS TUA) cohort [26]. Eight hundred and fifteen (815) older adults age 60 years and above were recruited via multi-stage random sampling from two states in Malaysia; Older adults with dementia, any known psychiatric problems, severe vision, speech and auditory problems, and who were non-ambulant were excluded at baseline.

A total of 49.1% of the participants (400) were successfully followed up at 5 years. Others, 4.8 and 46.1%, had passed away and refused to participate or were not contactable, respectively. Ethical approval (UKM1.21.3/244/NN-2018-145) and written informed consent for this study was obtained.

### Data collection

Data were collected by trained enumerators using a structured questionnaire and tests at the nearest community centres. The questionnaires used in this study have been validated and reported previously [26]. It consisted of socio-demography, medical, falls, psycho-social, lifestyle, dietary intake, blood pressure, anthropometry, physical fitness and functional status information. Baseline assessments were repeated at five years of follow-up. All methods were carried out in accordance with relevant guidelines and regulations. Participants were provided monetary incentives to cover their travelling expenses.

### Assessment of physical and cognitive function

#### Mild cognitive impairment (MCI)

Participants were categorized as having a mild cognitive impairment (MCI) if they had subjective memory complaint (either by participants or caregivers), objective memory impairment (at least 1.5 SD below the mean for either Rey Auditory Verbal Learning Test, RAVLT or digit span tests), without or very minimal functional limitations in basic activities of daily living (ADL) (at least 1.5 SD below the mean), maintained global cognitive function (Malay version of Mini-Mental State Examination, M-MMSE score of > 19 and not diagnosed to have dementia, reported by a doctor/physician at baseline. These criteria were benchmarked based on Petersen et al. [27] and Lee et al. [28]. Participants who did not fit into these criteria were categorized as normal.

#### Physical frailty

Physical frailty was assessed using Fried et al. [1] criteria and the cut-off points outlined in the Cardiovascular Health Study (CHS). The components of assessment consisted of; 1) unintentional weight loss of more than 5 kg in the prior years, 2) self-reported exhaustion or tiredness based on two items of the Centre of Epidemiologic Studies Depression scale (CES-D), 3) low physical activity assessed using the Physical Activity Scale for Elderly (PASE) [29], 4) weakness, measured using dominant hand grip strength (digital hand dynamometer; Jamar® Plus+, Patternson Medical, IL, USA); and 5) slowness, measured using the five-metre gait speed test.

#### Cognitive frailty (CF)

CF was categorised based on the presence of both physical (pre-frailty/frailty) and cognitive (subjective cognitive complaint; SCC/mild cognitive impairment; MCI) as reported in our previous study [13].

### Other outcome measures

#### Falls

At every follow up, including at the five years follow up, participants were asked if they had a fall in the past year. A fall in this study was defined to participants as “an event whereby a person inadvertently comes to rest on the ground or a lower level, excluding intentional change in position to rest on another object” [14]. Participants with a history of falls (one or more) in the past 5 years were classified as fallers, and those who did not were grouped as non-fallers.

#### Fall-related injuries

Participants who reported as having injurious falls (bruises, scars, bleeding or fractures) in the past five years were classified as the group with injuries, and those without any injuries was grouped into non-injury group.

#### Disability

Disability was assessed using the WHO Disability Assessment Schedule (WHODAS), that captures six major domains, namely, self-care, participation, cognition, mobility, getting along, and life activities. WHODAS 2.0, 12-item questionnaire with five-point Likert scale (0: none,1: some, 2: moderate, 3: severe and 4: very severe) assesses restriction in activities of daily living and social participation due to health problems for the past 1 month [30]. Levels of disability were categorised as following: 0 = no disability, 1 to 4 = mild disability, 5 to 9 = moderate disability, and 10 to 48 + more severe disability. In this study, participants were grouped into two groups, with (those categorised as severe disability) and without disability (all the remaining).

### Potential confounding factors

Multiple factors were taken into account as adverse outcomes that may be due the interactions of these factors occurring over an extended period of time. These factors have been reported in detail previously [26] and includes:

#### Socio-demographic information and medical history

This information consisted of age, gender, ethnicity, years of education, household income, living arrangement (living alone or with others), smoking status, and medical history.

#### Nutritional status and body composition

Anthropometric measurements (height, weight, waist circumference, mid-upper arm circumference; MUAC, and calf circumference) were measured. Body Mass Index (BMI, kg/m^2^) was calculated as body weight in kilograms divided by squared standing height in meters. Body composition was obtained using the Bio-electrical Impedance Analysis InBody S10 (Biospace, Seoul, Korea).

#### Laboratory analysis

A total of 20 ml fasting peripheral venous blood was withdrawn using a butterfly syringe by a trained phlebotomist for the biochemical analysis, which consisted of fasting blood sugar (FBS), Haemoglobin A1c (HbA1c), total cholesterol (TC), high-density lipoprotein (HDL), low-density lipoprotein (LDL), and albumin (ALB).

#### Psycho-social and functional assessments

Geriatric depression scale-15 (GDS) was used to assess potential depressive symptoms [31]; loneliness was evaluated using ‘three-item loneliness scale’ [32]; Medical Outcome Study Social Support Survey (MOSS) to assess social support; functional status was assessed using Instrumental Activities of Daily Living (IADL) [33].

#### Lifestyle activities

Lifestyle activities were measured using the adapted version of the Victoria Longitudinal Study-Activities Lifestyle Questionnaire (VLS-ALQ) which consisted of 26 lifestyle items divided into three main domains namely physical, mental and social activities [34].

#### Other cognitive function assessment

Global cognitive function was assessed using the Malay version of Mini-Mental State Examination (M-MMSE) and the Montreal Cognitive Assessment (MoCA) [35]; the Weschler Memory Scale-Revised (WMS-R) and the Digit Span Forward and Backward test was used to assessed attention and working memory; the Digit Symbol test was administered to measure the information processing speed; and the Rey Auditory Verbal Learning Test (RAVLT) for verbal learning and memory [36].

#### Other physical function tests

These included 2-min step test (endurance), chair sit and reach (lower body flexibility), chair stand test (lower body strength), timed-up-and-go (balance and mobility), back scratch test (upper body flexibility) and hand grip (upper body strength) based on senior fitness test [37].

#### Dietary intake

Dietary intake was obtained using the Dietary History Questionnaire (DHQ) [38] and analysed using Nutritionist Pro software.

### Statistical analysis

Descriptive statistics were performed to identify the differences in participants’ characteristics according to drop-out or non-dropout groups. The dependent variable was the participants in drop-out group as the reference variable, compared to non-dropout group. The cumulative incidence rates of the adverse consequences, including falls, fall-related injuries and disabilities, were calculated during the follow-up period according to participants’ frailty subtypes (PF and CF) and mild cognitive impairment (MCI) at baseline. Intergroup differences were estimated using the univariate logistic regression models. The incidence rates were calculated as the number of new cases of falls, injuries and disability divided by the total person-time observed between the two assessments. The number of at-risk participants and total person-time referred to participants with no falls, injuries and disability at baseline. The age-specific incidence rates were computed for 5-year age categories (60–64, 65–69, 70–74, 75–79, and 80 years and above) using a person-year analysis. An age-specific incidence rates curve was modelled.

To identify the confounding factors for all adverse outcomes, descriptive statistics were used to present characteristics of participants according to fallers or non-fallers; with injuries and without injuries; with disabilities and without disabilities The socio-demographic factors, medical history, nutritional assessments, clinical profile, laboratory analysis, psycho-social, functional status, cognitive and physical assessments, and dietary intake were compared between these two groups using a Chi-Square test (χ^2^) for categorical variables and independent t-test for continuous variables. The baseline characteristics of the participants were presented as mean and standard deviation for continuous data and as frequency and percentages for categorical data. The significant value was set at *p* < 0.05.

A hierarchical Binary Logistic Regression (BLR) analysis was performed to determine the confounding factors associated with each of the adverse consequences (falls, injuries, and disabilities) in a multivariate model. The BLR analysis was computed separately for every outcome due to the different confounding factors. First, all the significant variables (*p* < 0.05) from the univariate analysis were adjusted for multiple testing and classified into five different models: (1) socio-demographic and medical history; (2) nutritional status, clinical profile, laboratory analysis; (3) psycho-social and functional status; (4) physical and cognitive assessments and; (5) dietary intake. Next, variables that were significant (p < 0.05) in each model were included into the final binary logistic model as confounding factors for CF status. Adjusted odds ratios (Adj ORs) for the incidence of the adverse consequences (falls, injuries, and disability) and their 95% confidence intervals (95% CI) were estimated. Besides, we also derived a composite measure of MCI, PF and CF computed from the mean z scores converted from the raw score of the components in each group, with appropriate score reversals and higher scores indicating worse condition. These continuous measures were further analysed using the same hierarchical model as the categorical measures, where the Adj ORs and 95% CI were obtained. To account for potentially selective losses to follow-up, we used inverse probability treatment weighting (IPTW), as estimated by the propensity score and presented as weighted OR with 95% CI. All analyses were performed using IBM SPSS version 25.0 (IBM 25.0, Tokyo, Japan). In all analysis, *p* < 0.05 was considered to indicate statistical significance.

## Results

Out of 815 participants at baseline, 49.1% (400) of the participants were successfully followed up after 5 years for the incidence analysis. The baseline characteristics, cognitive and physical status of the drop-out (*N* = 415) and the non-dropout group (*N* = 400) are as presented in Table 1. Participants in the drop-out group were significantly older and living alone at the baseline assessment (*p* < 0.05).

**Table 1:** The baseline characteristic, physical and cognitive status of drop-out and non-dropout participants [presented as mean ± standard deviation (sd) or n (%)]

Parameters	***N*** = 815		***p*** Value
	Drop-out (n = 415)	Non-dropout (n = 400)	
Age, mean ± sd	69.03 ± 6.37	67.65 ± 5.26	0.001*
Gender:			
Male	192 (46.2)	180 (45.1)	0.410
Female	224 (53.8)	219 (54.9)	
Ethnic:			
Malay	160 (38.5)	162 (40.6)	0.567
Chinese & Indian	256 (61.5)	237 (59.4)	
Marital status:			
Single/divorced	134 (32.2)	91 (22.8)	0.003*
Married	282 (67.8)	308 (77.2)	
Education years, mean ± sd	5.93 ± 4.33	6.37 ± 4.57	0.160
Household income, mean ± sd	1725.18 ± 3347.61	1707.24 ± 2437.72	0.931
Social support (MOSS), mean ± sd	39.55 ± 14.01	36.88 ± 15.23	0.141
Living alone	59 (14.2)	30 (7.5)	0.002*
Smoking	99 (23.8)	89 (22.3)	0.619
Alcohol drinking	29 (7.0)	25 (6.3)	0.778
Diseases:			
Diabetes	129 (31.0)	99 (24.8)	0.051
Hypertension	216 (51.9)	193 (48.4)	0.327
Hypercholesterolemia	123 (29.6)	122 (30.6)	0.760
Heart disease	36 (8.7)	32 (8.0)	0.800
Mild Cognitive Impairment (MCI)	45 (10.8)	55 (13.8)	0.202
Frailty status (pre-frailty & frailty)	330 (79.3)	296 (74.2)	0.097
Cognitive frailty	167 (40.1)	157 (39.3)	0.830

*Significant at *p* < 0.05. Abbreviation: *MOSS* Medical Outcome Study Social Support Survey

**Fig. 1:**
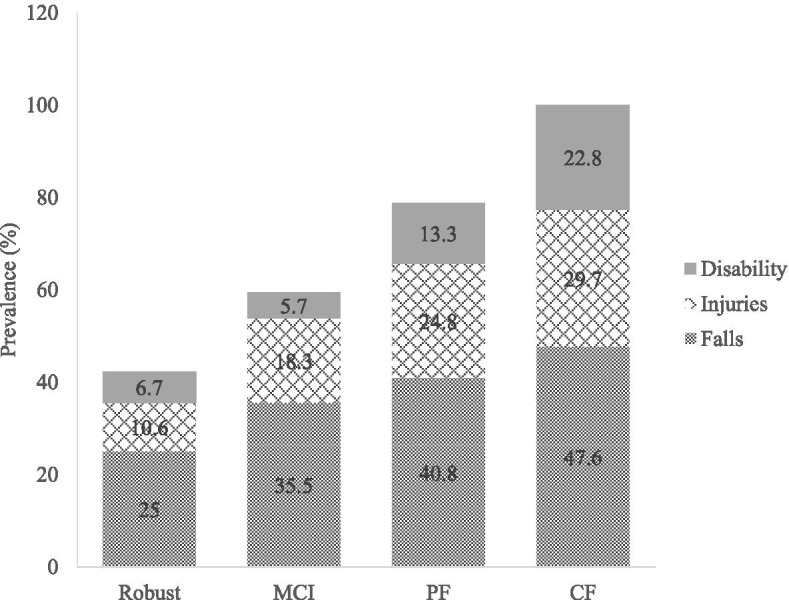
Prevalence of falls, injuries and disability after 5 years follow-up based on their physical and cognitive status at baseline

At baseline, there were 53 (13.3%), 144 (36.0%), and 158 (39.6%) participants with MCI, PF and CF respectively. The proportions of participants with the adverse consequences are as depicted in Fig. 1. CF group had the highest proportion of participants with incidence of falls (47.6%), injuries (29.7%), and disability (22.8%) (*p* < 0.05).

**Fig. 2:**
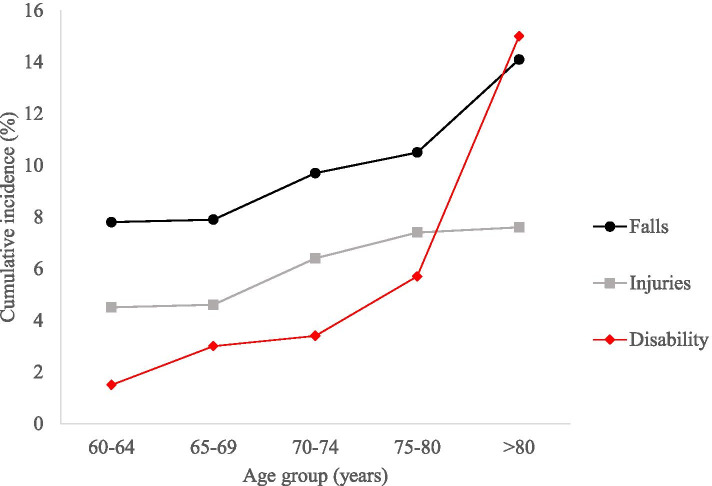
Cumulative incidence of falls, falls-related injuries and disability after 5 years follow up by age group of older adults

Participants were also divided based on age groups prior to determining the trend of the incidence rates for falls, injuries and disabilities. These age-specific cumulative incidence of falls, injuries and disabilities are as shown in Fig. 2. The overall incidence rate of falls after 5 years follow up were 7.8 per 100 person-years in the 60–64 year age group, 7.9 per 100 person-years in the 65–69 year age group, 9.7 per 100 person-years in the 70–74 year age group, 10.5 per 100 person-years in the 75–80 year age group to 14.1 per 100 person-years in the 80+ year age group. For falls-related injuries, the incidence rates were 4.5, 4.6, 6,4, 7.4 and 7.1 per 100 person-years in 60–64, 65–69, 70–75. 75–80, 80+ year age groups respectively. The incidence rate for disabilities were 1.5 per 100 person-years in 60–64, 3.0 per 100 person-years in the 65–69, 3.4 per 100 person-years in the 70–74, 5.7 per 100 person-years in the 75–80 and 15.0 per 100 person-years in the 80+ years age groups.

Possible confounding factors for the falls, injuries and disabilities were grouped according to each adverse consequence (Table 2). For falls, the significant factors were age, gender, years of education, percentage of body fat, fat-free mass, skeletal muscle mass, fasting blood sugar, HbA1c, social support, loneliness, WHODAS, energy and carbohydrate intake (*p* < 0.05). Whereas age, living alone, BMI, calf circumference, waist circumference, hip circumference, mid-upper arm circumference, fat-free mass, skeletal muscle mass, diastolic blood pressure, WHODAS, MMSE and carbohydrate intake (*p* < 0.05) were significantly different between those with and without injuries groups. For disabilities, the significant variables included age, years of education, household income, BMI, waist circumference, body fat percentage, fat mass, fat-free mass, skeletal muscle mass, geriatric depressive syndrome, social support, IADL, ALQ, fibre, riboflavin and niacin intake (*p* < 0.05). It is noteworthy that all cognitive and physical assessments were found to be significantly different between those with and without disabilities groups (*p* < 0.05).

Table 3 shows older adults with CF appeared to have the highest risk of falls, injuries, and disability. Both PF (Adj OR = 2.88, 95% CI: 1.19–6.99, *p* < 0.05; Weighted OR = 2.50, 95% CI: 1.43–4.38, *p* < 0.05) and CF (Adj OR = 2.98, 95% CI: 1.78–4.99, *p* < 0.05; Weighted OR = 2.68, 95% CI: 1.71–4.20, *p* < 0.05) significantly predicted the incidence of falls after adjusting for age, gender, years of education, waist circumference, social support and loneliness. Similarly, after adjusting for covariates (age, years of education, body mass index, arm circumference, MMSE) the risk of injuries were significantly increased with the presence of both PF (Adj OR = 3.04, 95% CI: 1.75–5.28, *p* < 0.05; Weighted OR = 2.21, 95% CI: 1.11–4.38, p < 0.05) and CF (Adj OR = 3.06, 95% CI: 1.23–7.60, p < 0.05; Weighted OR = 1.71, 95% CI: 1.03–2.84, p < 0.05). In addition, PF (Adj OR = 4.99, 95% CI: 1.11–22.57, p < 0.05; Weighted OR = 2.03, 95% CI: 1.16–3.56, p < 0.05) and CF (Adj OR = 5.17, 95% CI: 1.11–24.21, p < 0.05; Weighted OR = 3.57, 95% CI: 1.35–9.42, p < 0.05) significantly predicted the incidence of disability among older adults after adjusting for age, years of education, waist circumference, chair stand test, back scratch test, social support and depression. In addition, the continuous measures of PF and CF showed a significant association with incident of falls, injuries and disability assessed in the hierarchical models (p < 0.05).

**Table 3:** Potential predictors (cognitive and physical statuses at baseline) for the incidence of falls, injuries and disability at five years

Predictor of interest (Cognitive and physical statuses at baseline)	***Categorical analysis***			***Continuous analysis (z-score)***		
	B	Adjusted OR (95% CI)	Weighted OR (95% CI)	B	Adjusted OR (95% CI)	***p*** Value
**Falls**						
Cognitive impairment	0.43	1.54 (0.74–3.17)	1.33 (0.72–2.49)	0.11	1.12 (0.90–1.40)	0.249
Physical frailty	1.06	2.88 (1.19–6.99)^a^	2.50 (1.43–4.38)^a^	0.23	1.26 (1.07–1.49)^a^	0.003
Cognitive frailty	1.12	2.98 (1.78–4.99)^a^	2.68 (1.71–4.20)^a^	0.35	1.42 (1.12–1.80)^a^	0.003
**Falls-related injuries**						
Cognitive impairment	−0.48	0.62 (0.22–1.72)	1.56 (0.75–3.25)	0.06	1.06 (0.83–1.36)	0.637
Physical frailty	1.12	3.04 (1.75–5.28)^b^	2.21 (1.11–4.38)^b^	0.33	1.39 (1.07–1.80)^b^	0.013
Cognitive frailty	1.11	3.06 (1.23–7.60)^b^	1.71 (1.03–2.84)^b^	0.19	1.21 (1.01–1.45)^b^	0.037
**Disability**						
Cognitive impairment	0.28	1.33 (0.18–10.01)	1.45 (0.69–3.06)	0.13	1.14 (0.85–1.54)	0.348
Physical frailty	1.61	4.99 (1.11–22.57)^c^	2.03 (1.16–3.56)^c^	0.31	1.36 (1.09–1.70)^c^	0.002
Cognitive frailty	1.64	5.17 (1.11–24.21)^c^	3.57 (1.35–9.42)^c^	0.48	1.62 (1.17–2.23)^c^	0.004

^a^indicated *p* < 0.05, after adjusted for age, gender, years of education, waist circumference, MOSS, loneliness

^b^indicated *p* < 0.05, after adjusted for age, years of education, body mass index, arm circumference, MMSE

^c^indicated p < 0.05, after adjusted for age, years of education, waist circumference, chair stand test, back scratch test, MOSS, GDS

**Table 2:** The baseline attributes of the participants detected with and without adverse health outcomes at the 5-years follow-up (presented as mean ± standard deviation (sd) or number of participants (%)]

Parameters	Fall at five years (***N*** = 400)			Fall-related Injuries at five years (N = 400)			Disability at five years (N = 400)		
	Faller (***n*** = 136)	Non-faller (***n*** = 264)	***p*** Value	Injured (***n*** = 97)	Non-injured (***n*** = 303)	***p*** Value	Disabled (***n*** = 61)	Non-disabled (***n*** = 339)	***p*** Value
Age	68.98 ± 6.17	67.63 ± 5.33	<.001*	69.37 ± 6.55	67.55 ± 5.25	.004*	70.66 ± 6.21	67.10 ± 4.90	<.001*
Sex:									
Men	45 (33.1)	134 (51.3)	<.001*	36 (37.1)	143 (47.2)	.085	23 (37.7%)	159 (46.6%)	.211
Women	91 (66.9)	130 (48.7)		61 (62.9)	160 (52.8)		38 (62.3%)	180 (53.4%)	
Ethnic:									
Malay	49 (36.0)	113 (42.9)	.197	35 (36.1)	118 (41.8)	.351	26 (42.6%)	136 (39.9%)	.777
Non-Malay	87 (64.0)	151 (57.1)		62 (23.1)	185 (58.2)		35 (57.4%)	203 (60.1%)	
Education (years)	5.20 ± 3.98	5.72 ± 4.08	.048*	5.60 ± 4.57	6.42 ± 4.52	.113	4.02 ± 3.47	6.77 ± 4.62	<.001*
Living alone	13 (9.6)	17 (6.5)	.318	15 (15.5)	25 (7.1)	.015*	8 (13.1%)	21 (6.2%)	.062
Smoker	29 (21.3)	60 (23.0)	.800	24 (24.7)	80 (22.6)	.684	17 (27.9%)	71 (20.8%)	.240
Household income	1285.83 ± 1856.91	1442.01 ± 2797.75	.296	1249.90 ± 1821.05	1782.34 ± 2536.02	.055	878.57 ± 1119.84	1852.85 ± 2576.85	<.001*
Health Conditions:									
Diabetes	38 (27.9)	60 (23.0)	.327	27 (27.8)	86 (24.3)	.509	20 (32.8)	80 (23.7)	.149
Hypertension	68 (50.0)	123 (47.1)	.598	46 (47.4)	172 (48.6)	.909	34 (55.7)	157 (46.4)	.211
Hypercholesterolemia	48 (35.3)	73 (28.0)	.137	34 (35.1)	106 (29.9)	.386	19 (31.1)	102 (30.2)	.881
Heart Disease	15 (11.0)	16 (6.1)	.113	11 (11.3)	26 (7.3)	.212	9 (14.8)	23 (6.8)	.068
Nutritional assessments									
BMI (kg/m^2^)	25.76 ± 4.18	25.44 ± 4.22	.386	24.21 ± 3.84	25.67 ± 4.11	.002*	26.60 ± 4.13	25.31 ± 4.02	.023*
CC (cm)	33.48 ± 3.64	33.67 ± 3.52	.418	33.29 ± 3.33	34.55 ± 3.54	.002*	34.13 ± 3.18	34.48 ± 3.52	.469
WC (cm)	88.03 ± 10.68	88.14 ± 10.51	.868	86.85 ± 11.09	89.60 ± 10.41	.024*	92.94 ± 9.36	88.73 ± 10.52	.004*
MUAC (cm)	28.59 ± 3.25	28.58 ± 3.18	.949	27.63 ± 3.10	29.19 ± 3.27	<.001*	29.38 ± 3.22	29.01 ± 3.12	.396
Body composition									
% Body Fat	40.32 ± 10.13	38.93 ± 10.49	.039*	38.28 ± 9.99	38.52 ± 10.13	.838	43.19 ± 10.28	37.91 ± 9.89	<.001*
Fat mass (kg)	25.00 ± 9.28	24.46 ± 8.81	.359	22.97 ± 9.15	24.90 ± 8.76	.057	27.91 ± 8.69	24.25 ± 8.72	.003*
Fat free mass (kg)	35.76 ± 7.01	37.42 ± 8.13	.001*	35.74 ± 7.47	38.87 ± 8.12	.001*	36.03 ± 7.20	38.80 ± 8.23	.014*
Skeletal muscle mass (kg)	18.93 ± 4.17	19.94 ± 4.86	.001*	18.92 ± 4.47	20.81 ± 4.86	.001*	19.10 ± 4.30	20.77 ± 4.93	.013*
Laboratory analysis									
FBS (mmol/l)	5.79 ± 1.55	6.05 ± 1.96	.038*	5.69 ± 1.45	6.03 ± 2.23	.222	43.19 ± 10.28	37.91 ± 9.89	.866
HbA1c (%)	13.71 ± 1.93	14.24 ± 2.19	.001*	14.18 ± 2.81	14.57 ± 2.67	.271	14.20 ± 2.24	14.54 ± 2.67	.406
Total cholesterol (mmol/l)	5.37 ± 1.14	5.39 ± 1.07	.780	5.21 ± 1.01	5.19 ± 1.00	.899	5.00 ± 1.02	5.22 ± 1.01	.159
HDL (mmol/l)	1.42 ± 0.36	1.40 ± 0.34	.327	1.52 ± 0.38	1.44 ± 0.37	.109	1.46 ± 0.37	1.46 ± 0.38	.978
LDL (mmol/l)	3.29 ± 1.05	3.33 ± 0.98	.620	3.04 ± 0.93	3.10 ± 0.91	.610	5.00 ± 1.02	5.22 ± 1.01	.192
Albumin (g/l)	42.76 ± 2.41	42.94 ± 2.53	.313	42.68 ± 2.65	43.05 ± 2.75	.303	42.38 ± 2.40	43.07 ± 2.73	.093
Psychosocial and functional status:									
GDS	2.69 ± 2.31	2.43 ± 2.14	.084	2.73 ± 2.43	2.58 ± 2.29	.579	4.43 ± 3.48	2.52 ± 2.67	<.001*
MOSS	37.89 ± 14.95	40.24 ± 14.84	.017*	36.57 ± 16.13	37.50 ± 15.19	.603	30.39 ± 16.89	38.18 ± 14.53	<.001*
Loneliness	3.34 ± 1.06	3.17 ± 0.74	.003*	3.29 ± 0.91	3.21 ± 0.84	.421	3.34 ± 1.05	3.20 ± 0.79	.022*
IADL	12.76 ± 2.00	12.75 ± 1.84	.904	13.13 ± 1.52	13.13 ± 1.59	.977	12.77 ± 1.76	13.21 ± 1.53	.046*
WHODAS	6.28 ± 8.69	4.54 ± 6.55	.010*	7.03 ± 9.17	5.37 ± 7.52	.001	8.66 ± 8.16	4.50 ± 7.03	<.001*
ALQ	39.25 ± 10.35	39.82 ± 10.28	.404	39.91 ± 10.52	41.99 ± 9.89	.124	37.08 ± 9.82	42.71 ± 9.91	<.001*
Cognitive assessment									
MMSE	23.45 ± 4.64	23.86 ± 4.19	.153	23.29 ± 5.56	24.66 ± 4.08	.008*	22.36 ± 5.41	24.85 ± 3.97	<.001*
MoCA	19.31 ± 5.56	19.82 ± 5.37	.156	20.67 ± 5.32	23.29 ± 5.56	.130	18.05 ± 5.57	21.08 ± 5.15	<.001*
Span Digit	7.57 ± 2.48	7.73 ± 2.32	.321	7.60 ± 2.53	8.16 ± 2.47	.052	7.67 ± 2.32	8.14 ± 2.52	.154
RAVLT	40.12 ± 9.69	40.42 ± 9.54	.640	39.84 ± 9.49	40.64 ± 10.36	.499	37.17 ± 9.31	41.01 ± 10.42	.008*
Digit symbol	5.12 ± 2.58	5.39 ± 2.73	.122	5.72 ± 3.01	6.21 ± 3.21	.193	4.68 ± 2.00	6.43 ± 3.27	<.001*
Fitness test:									
2-min step test	63.83 ± 26.57	66.13 ± 22.89	.156	67.71 ± 28.14	69.20 ± 24.11	.606	55.43 ± 28.69	70.83 ± 23.65	<.001*
Chair stand test	10.35 ± 3.00	10.49 ± 2.97	.461	10.48 ± 3.40	10.89 ± 3.09	.257	9.20 ± 2.67	11.18 ± 3.08	<.001*
Chair sit and reach test	0.21 ± 11.73	− 0.44 ± 10.75	.378	1.29 ± 9.79	1.94 ± 9.85	.570	4.48 ± 10.22	1.55 ± 9.76	.034*
TUG test	10.43 ± 2.75	10.18 ± 2.61	.156	9.60 ± 2.78	9.46 ± 2.48	.633	10.76 ± 2.61	9.20 ± 2.41	<.001*
Back scratch test	14.09 ± 12.53	13.66 ± 11.95	.599	13.80 ± 11.15	13.70 ± 12.55	.946	18.67 ± 13.12	12.65 ± 11.97	<.001*
MCI	15 (11.1%)	38 (14.4%)	.355	11 (10.1)	49 (14.4)	.161	35 (57.4%)	124 (36.5%)	.039*
Physical frailty	67 (49.6%)	100 (38.0%)	.032*	13 (11.9)	19 (5.6)	.025*	3 (4.9%)	50 (14.7%)	.003*
Cognitive frailty	82 (60.3%)	95 (36.4%)	<.001*	52 (47.7)	125 (36.8)	.028*	36 (59.0%)	124 (36.5%)	.002*
Nutrient intake									
Energy (kcal)	1633 ± 4.71	1698 ± 490	.047*	1608 ± 436	1656 ± 432	.345	1549 ± 438	1658 ± 427	.076
Protein (g)	70.30 ± 22.86	71.91 ± 21.72	.283	69.16 ± 22.78	69.70 ± 20.09	.828	68.25 ± 19.78	69.92 ± 21.10	.576
Carbohydrate (g)	217.67 ± 74.41	232.94 ± 80.85	.004*	205.08 ± 69.70	223.10 ± 70.92	.032*	202.29 ± 68.81	221.53 ± 69.56	.053
Fat (g)	53.27 ± 20.84	52.82 ± 18.86	.730	56.69 ± 19.71	53.36 ± 17.86	.125	51.79 ± 18.64	54.13 ± 18.06	.368
Fibre (g)	4.26 ± 2.83	4.02 ± 2.42	.155	4.66 ± 2.88	4.65 ± 2.57	.976	4.06 ± 2.13	4.81 ± 2.81	.022*
Vitamin C (mg)	116.08 ± 82.15	124.47 ± 88.82	.144	134.07 ± 77.29	134.40 ± 86.99	.973	125.42 ± 88.04	136.29 ± 86.91	.383
Vitamin D (μg)	0.30 ± 0.99	0.27 ± 0.81	.696	0.38 ± 0.84	0.34 ± 0.99	.742	0.24 ± 0.87	0.36 ± 0.99	.360
Vitamin E (mg)	14.45 ± 68.58	8.58 ± 41.43	.105	7.51 ± 19.83	5.96 ± 14.02	.399	8.04 ± 24.58	6.15 ± 14.37	.419
Thiamin (mg)	1.26 ± 2.48	1.56 ± 3.81	.148	1.10 ± 2.06	1.51 ± 3.11	.235	1.64 ± 3.33	1.54 ± 3.31	.836
Riboflavin (mg)	1.23 ± 0.50	1.24 ± 0.47	.732	1.25 ± 0.47	1.25 ± 0.45	.987	1.11 ± 0.43	1.29 ± 0.46	.006*
Niacin (mg)	10.51 ± 4.59	10.47 ± 3.76	.879	10.63 ± 4.82	10.04 ± 3.47	.198	9.26 ± 2.82	10.39 ± 4.00	.040*
Sodium (mg)	1447.62 ± 564.41	1517.57 ± 1080.13	.291	1421.61 ± 785.88	1443.61 ± 902.77	.830	1739.82 ± 1310.0	1422.52 ± 798.02	.079
Calcium (mg)	512.31 ± 424.24	529.64 ± 234.93	.281	517.19 ± 224.21	509.32 ± 220.00	.764	480.69 ± 215.99	515.61 ± 223.39	.272
Iron (mg)	13.53 ± 5.42	13.81 ± 5.19	.436	13.47 ± 5.18	13.65 ± 4.77	.749	13.24 ± 4.73	13.76 ± 4.85	.451
Zinc (mg)	3.63 ± 1.81	3.75 ± 2.00	.334	3.59 ± 1.58	3.77 ± 1.88	.527	3.50 ± 1.88	3.75 ± 1.87	.359

*Significant at *p* < 0.05. Notes: CF cognitive frailty; *BMI* Body Mass Index; *CC* Calf circumference; *WC* Waist circumference; *HC* Hip circumference; *MUAC* Mid-upper arm circumferences; *FBS* Fasting Blood Sugar; *ALQ* activities of lifestyle questionnaire; *IADL* instrumental activities of daily living; *GDS* Geriatric Depression Scale; *WHODAS* WHO Disability Assessment Schedule; *MOSS* Medical Outcome Study Social Support Survey; *MMSE* Mini-Mental State Examination; *MoCA* Montreal Cognitive Assessments; *RAVLT* Rey Auditory Verbal Learning Test; *TUG* Timed-up-and-Go test; and *sd* standard deviation

**Fig. 3:**
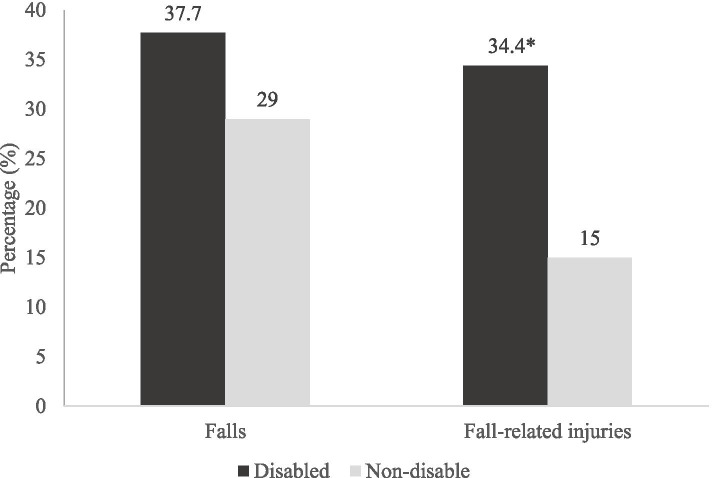
The percentage of falls and fall-related injuries in the group with and without disability at 5 years follow up. * indicated p < 0.05 using the chi-square test

Figure 3 shows the percentage of falls and fall-related injuries in the group with and without disability. Even though there is no significant association between falls and disability (*p* > 0.05), the findings indicated the prevalence of fallers in the disability group was higher compared to those without the disability. Besides, the prevalence of fallers with injuries was significantly higher in the disability group in comparison to those without disability (*p* < 0.05).

## Discussion

The aim of our present prospective cohort study was to determine the predictive ability of cognitive frailty (CF) on its adverse consequences consisting of falls, falls-related injuries and disability among community-dwelling older adults. The prevalence of CF based on the operational definition of CF among this cohort at baseline was 39.6% [39]. The study results indicated that CF is a robust predictor of falls, falls-related injuries and disability in comparison to physical frailty (PF) solely. These findings may be the first empirical evidence regarding the relationship between CF and adverse outcomes (falls, related injuries and disability) among community-dwelling older adults in Malaysia.

These findings are expected as CF is the combination of both PF and MCI. Its combination was deduced to intensify the risk of adverse consequences among older adults [40]. There may be a possibility of the overall general health of older adults being affected by the interaction of both cognitive and physical impairments modulating other bio-psychosocial issues that led to a heightened risk for falls, injuries and disability. Physical and cognitive decline are associated and may interact synergistically [5]. Although both PF and MCI on its own present a risk, the risk of adverse consequences among older adults with PF was lesser when compared to CF. MCI on its own without PF did not appear to be a significant determinant of adverse consequences in our study.

The estimated age-specific incidence rate projection of falls, injuries, and disability among the older adults in our study rose steadily with increasing age. Notably, falls and disability incidence rate was seen to be increasing exponentially at age 75 and above. In contrast, fall-related injuries seemed to reach a plateau with increasing age. Among the possible reasons for this plateauing of the curve for injuries could be due to reduced mobility and risk-taking behaviours among the affected older adults at this age or because there is a small proportion of older adults living up to this age [41]. Fear of fall (FOF) is common among older adults whether or not they have sustained a fall, resulting in a reduction in activity that they are still capable of performing [42]. Inactivity due to FOF leads to deconditioning and loss of muscle strength in older adults. Inversely, FOF could also result in older adults taking extra care and precautions during activities and avoiding any dangerous tasks, possibly preventing falls and fall-related injuries [43].

In our study, the falls incidence rate consistently increased with the incidence rate of injuries across the age groups. Literature supports the fact that there is an increased falls risk with advancing age [41]. Similarly, in the Malaysian context, advancing age was shown to be a risk factor for fall risk among the older population [17, 44]. Prevalence and incidence of falls are known to increase with ageing mainly due to declining performance across multiple systems, which includes physical and cognitive functions [4, 15, 45]. Age-related changes such as decreased muscle strength, postural instability and cognitive impairment justify the correlation of age and fall risk [4, 43]. Besides, a common serious injury as a result of a fall are fractures [44]. There is evidence that decreases in bone mineral density (BMD) increased the risk of falls and fractures specifically in older people with advancing age, sarcopenia, low level of physical exercise or activity, impaired mobility and frailty [46, 47].

Falls and falls-related injuries were reported to have an association with the occurrence of disability, loss of independence and increased mortality [48, 49]. In accordance with previous studies, our study findings demonstrated that the incidence rate of disability not only increased across the age groups but was higher among older adults with falls and fall-related injuries. It is noteworthy that ageing itself was associated with increased prevalence of having diseases, particularly chronic diseases, which is a known contributor to the occurrence of disability as well [50]. The occurrence of falls and injuries among older adults could further aggravate the progression to living with disabilities with a higher likelihood of long-term nursing home admissions [48]. Therefore, our study findings highlight the need for overall falls and injurious falls prevention efforts in view of alleviating the burden of disability among older adults.

Moreover, PF and CF were demonstrated as predictors of fall incidence after controlling for the associated confounding factors. Similar findings were reported in recent cross-sectional and longitudinal studies [22, 25]. Slow gait speed, the main feature of PF, is associated with cognitive deficits in processing speed, attention and executive functions, predisposing to increase the risk of falls among older adults [3, 4, 51]. Moreover, older adults with CF may have declined reaction time with possible visual or hearing impairments, leading to postural hence more susceptible to falls [52].

We have also previously reported that there is a relationship between CF, depressive symptoms and inadequate of vitamin D intake in older adults [12]. For instance, symptoms that are commonly seen in the geriatric population with depression, such as poor appetite, weight loss, malnutrition and specifically nutritional deficiencies in vitamin D and folate which may have a direct impact on falls incidence [53]. Hence, falls prevention strategies should take into account other issues such as depression and nutritional deficiencies, more so for older adults with CF.

In fact, the presence of cognitive impairment among physically frail individuals renders them to often engage in risky activities, increasing their fall risk and posing additional risks of injuries such as fractures [54]. Their frail condition may result in the inability to safely break a fall or avoid injuries after a fall [22]. In addition, the insufficient vitamin D among cognitively frail older adults may lead to an increase in bone loss and, as a consequence, increase falls risk, related injuries and disability [12, 47]. This was evident in our present study, whereby a higher percentage of older adults with falls and related injuries were categorised in the group with disability.

In our study, both PF and CF were demonstrated to have a predictive ability on the occurrence of severe disability among older adults. This finding is further supported by the study by Tsutsumimoto et al. [24], where CF was found to be associated with incidence of disability compared to PF or cognitive impairment on its own. Older adults with PF or even prefrailty have an increased risk of being predisposed to disability [8]. Note that both PF and cognitive impairments are associated with a higher level of inflammatory markers, indicating that CF may represent a state of increased inflammation [55]. Elevated inflammation markers in older adults with CF could trigger the incidence of disability by accelerating the loss of muscle mass and impairing muscle tissue regeneration following injury [55, 56]. Thus, early prevention and management of CF in older adults are vital to prevent the incidence of adverse health outcomes, namely disability.

The main limitation of this study is the high drop-out rate of participants at the five-year follow-up. This could contribute to an under-representation of the study population. However, this is a common problem in longitudinal studies involving older adults. Besides, the participants in the drop-out group were older and living alone, as demonstrated in our present study and an in a previous report [20]. The IPTW weighted results were also similar to unweighted results, indicating that the high drop-out rate in this study did not impact the predictive ability of PF and CF on adverse health outcomes. Additionally, certain information was self-reported in nature, such as medical conditions and lifestyle information. Despite these potential limitations, this longitudinal study findings provided an insight of a dynamic and complex cause-and-effect relationship between MCI, PF and CF as adverse outcomes of ageing among Malaysian older adults after adjustments of confounding factors. Our study results are also based on a comprehensive set of parameters and clinical outcomes that may identify the pre-disposing factors of these adverse outcomes using simple yet valid tools. Besides, the inclusion of pre-frailty in the CF operational definition has increased the possibility of detecting larger number of older adults with CF when compared to the previous definition. Understanding the potential of CF as a robust predictor of these adverse events could facilitate in identifying older people who might benefit from early prevention and interventions in the community, such as the ‘WE-RISE” multi-domain intervention [57].

## Conclusion

In conclusion, both CF and PF were predictors of fall, fall-related injuries and disability in older adults, with CF being more robust of the two. These findings could guide healthcare professionals and policymakers in the planning of health promotion that is specifically tailored for older adults with CF. Possible early multi-domain preventive and management strategies of CF that contribute to a detrimental cascade of adverse events are required to decrease further functional decline and promote independence in older adults.

## Data Availability

The datasets used and/or analysed during the current study are available from the corresponding author on reasonable request.
